# Do human embryos have the ability of self-correction?

**DOI:** 10.1186/s12958-020-00650-8

**Published:** 2020-10-06

**Authors:** Raoul Orvieto, Chen Shimon, Shlomit Rienstein, Anat Jonish-Grossman, Hagit Shani, Adva Aizer

**Affiliations:** 1 Infertility and IVF Unit, Department of Obstetrics and Gynecology, Chaim Sheba Medical Center (Tel Hashomer), 56261 Ramat Gan, Israel; 2 Sackler School of Medicine, Tel Aviv University, Tel Aviv, Israel; 3 The Tarnesby-Tarnowski Chair for Family Planning and Fertility Regulation, Sackler Faculty of Medicine, Tel-Aviv University, Tel Aviv, Israel; 4 Danek Gertner Institute of Human Genetics, Sheba Medical Center, 56261 Ramat-Gan, Israel

**Keywords:** Self-correction, PGT, Human embryo, Blastocyst, Mosaicism

## Abstract

Human embryogenesis frequently coinciding with cell division mistakes contributing to pervasive embryonic aneuploidy/mosaicism. While embryo self-correction was elegantly demonstrated in mouse models, human studies are lacking. Here we are witness to human embryos ability to eliminate/expel abnormal blastomeres as cell debris/fragments. Each blastocyst and its corresponding debris were separated and underwent whole genome amplification. Seven of the 11 pairs of blastocysts and their corresponding cell debris/fragments revealed discordant results. Of the 9 euploid blastocysts, four showed euploid debris, while in the others, the debris were aneuploid. In the remaining pairs, the debris showed additional aneuploidy to those presented by their corresponding blastocyst. The observed ability of human embryos to self-correction doubts many invasive and non-invasive preimplantation testing for aneuploidy at the blastocyst stage, rendering high rate of false positive (discarding “good” embryos) by identifying the cell-free DNA originated from the expelled cell debris, as aneuploidy/mosaic blastocyst.

## Introduction

The first stages of human embryogenesis are characterized by rapid cell proliferation, which frequently coinciding with cell division mistakes, generating changes in chromosome content, e.g. aneuploidy [1–3]. Oocyte meiotic aneuploidies may result from non-disjunction or premature separation of a chromosome into sister chromatids [4], and appears in the whole embryo’s cells [4, 5]. Along with errors in meiosis, mitotic errors during post-zygotic cell division contribute to pervasive aneuploidy in human embryos [6]. Mitotic mistakes are common, with the highest occurrence through the first three cleavages after fertilization [7]. Consequently, the majority of the human preimplantation embryos show aneuploidies that appears mainly as a diploid–aneuploid mosaicism. As with meiotic errors, mitotic mistakes also decrease with embryonic development. This was elegantly observed while re-analyzing blastocyst-stage embryos, which were detected as aneuploid at the cleavage stage (Day-3) [8–10].

Mosaicism has been reported in, as high as 50% of cleavage- and blastocyst-stage embryos derived from IVF [11]. Preliminary studies suggest that “mosaic” embryos display low rates of concordance between multiple trophectoderm (TE) biopsies. Moreover, “mosaic” embryos demonstrate increased cell proliferation and cell death in comparison to euploid embryos, both observations suggestive of significant self-correction abilities of embryos [12–14].

Santos et al. have suggested that embryo self-correction may result from increased aneuploidy cells death, or decreased cell division rate [15]. These suggested mechanisms might be supported by observations of aneuploid blastomeres leaving the blastocyst following the activation of apoptotic pathways [16]. Daughtry et al. [17] have demonstrated that Rhesus embryos overcome chromosome instability during preimplantation development by encapsulation of chromosome-containing cellular fragments into micronuclei and their elimination via cellular fragmentation. In their elegant mice study, Bolton et al. shed additional light onto the embryo’s ability to self-correct [14]. Treating mouse embryos with a spindle assembly checkpoint inhibitor during the 4- to 8-cell division, the authors generated chimeric embryos with euploid and aneuploid cells, which they followed via live-embryo imaging and single cell tracking. They found that aneuploid cells in the fetal lineage (i.e., inner cell mass producing the fetus) were eliminated by apoptosis, while those in the placental lineage (i.e., the TE) did show proliferative defects though survived. In a recently published study from the same group, Singla et al. [18] have demonstrated that aneuploid cells are preferentially eliminated from the embryonic lineage in a p53-dependent process involving both autophagy and apoptosis before, during and after implantation. It might be therefore argued, that early embryos development could be controlled by the establishment of a cell death program to ensure the elimination of damaged cells [19], while maintaining an optimal balance between survival and apoptotic signals.

Studies of cell death and survival in preimplantation embryos use mainly, mice models, aiming to overcome the ethical concerns limiting human embryos research. However, species specific differences might limit the extrapolation of results from mouse to human embryos. For example, Haouzi et al. [20] showed that while mouse and human embryos express components of the apoptotic and survival pathways during early embryonic development, their expression profile differs.

Recently, we presented a case of a cleaving human embryo from one to- two cells, with one cell hatching out from the zone pellucida, and thereafter their development into two blastocysts [21]. The two blastocysts were separated and sent to a comprehensive molecular analysis (polymorphic markers and array-CGH), demonstrating identical twins. The embryo in the zona pellucida showed normal balanced chromosomal profile, while the embryo that was expelled from the zona pellucida showed unbalanced chromosomal profile. In the present case, we were evident of ‘normalization’ or ‘self-correction’ of the chromosomally abnormal human preimplantation embryo by splitting in to two embryos, already in the first cell-division.

**Fig. 1:**
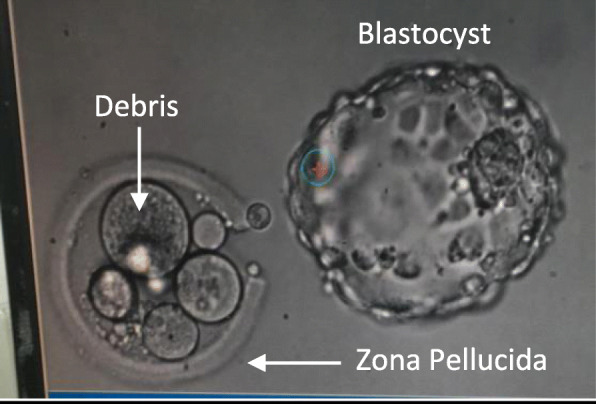
Day 5 hatched blastocyst and its original zona pellucida containing leftovers of cell debris

Even more intriguing, is the ability of human “mosaic” blastocysts to implant and develop to a viable pregnancy, though, with higher rates of miscarriage than euploid blastocyst [22]. One explanation for this ‘self-correction’ ability of an abnormal mosaic embryo to actually develop into a viable pregnancy, has been the natural apoptosis of abnormal cells. Of notice, finding direct evidence for any corrective mechanisms during early human development is extremely challenging. To overcome these limitations, we used affected / discarded human embryos undergoing preimplantation genetic testing for monogenic aberrations (PGT-M) and gender related disorders. In our PGT-M program, DNA extraction is obtained during the cleavage stage, where one blastomere from Day 3 embryo is extracted and undergo genetic diagnosis. In our routine clinical practice, following Day-3 biopsy, healthy embryos are transferred on Day-4 or 5, and the affected embryos are discarded. When these embryos were cultured until Day-5-6, we noticed that some of the blastocysts expel cell debris/ fragments within the zona pellucida (Fig. 1).

Prompted by this observation, we sought to examine the DNA content of these debris and their corresponding blastocyst (affected embryos that their day-3 blastomere biopsy revealed single-gene defect, and were donated for research by the couples), aiming to prove the hypothesis, that human embryos have the ability to self- correction and can eliminate/expel abnormal blastomere (the debris).

## Material and methods

**Fig. 2:**
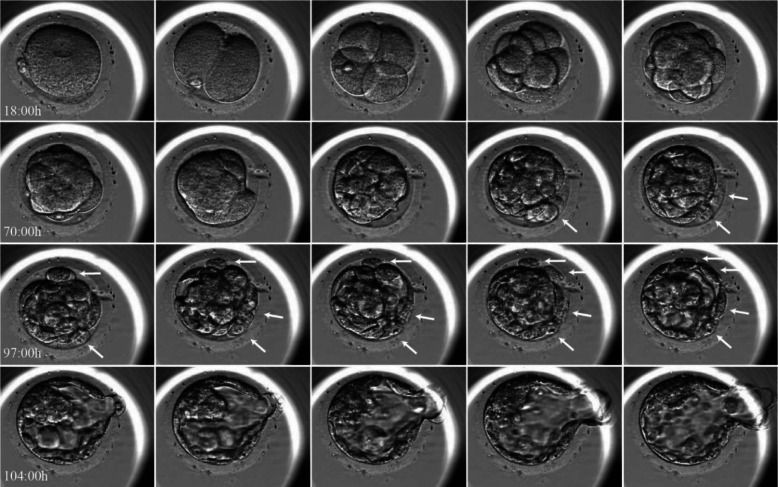
Time-lapse EmbryoScope™ photography of embryo expelling cell debris/cell fragments within the zona pellucida. Arrows pointing the cell debris

High-quality blastocysts [23], in which their Day-3 blastomere biopsy revealed an affected embryo with single-gene defect, were donated by couples undergoing PGT-M treatment at the Sheba Medical Center. Only blastocysts cultured in closed system using the time-lapse EmbryoScope™ incubator, that following hatching leaved cell debris/fragments within the zona pellucida were analyzed (Fig. 2 and Additional file 1). Each blastocyst and its corresponding debris were separated and underwent whole genome amplification (WGA).

### WGA

Full-genomic amplification of the DNA was carried out by WGA-PCR PicoPlex SingleCell WGA Kit (Rubicon Genomics) [24]. The quality and quantity of DNA received during amplification were controlled by electrophoresis using 1% agarose gel.

### Array-CGH

WGA products were processed referring to the protocol of Agilent oligonucleotide array-based CGH for single cell G4410–90003 Revision B0, October 2018. These products were fluorescently labelled with controls (Human Reference DNA Female/Male) according to the instructions of SureTag Complete DNA Labeling Kit (Agilent technologies, CA, USA), and then competitively hybridized to G9500A GenetiSure Pre-Screen Complete kit (8 × 60) Agilent technologies, CA, USA) [25].

### Interpretation of array-CGH results

Subsequent data analysis was performed according to the manufacturer recommended single cell analysis method. We only reported whole chromosome trisomies or monosomies.

The study required no modification of patient’s routine follow-up or treatment. Informed consent was obtained from all patients before participation in the study, and the study was approved by our Institutional Clinical Research Committee (IRB SMC-19-6140).

## Results

Twenty women (age 33.7 + 4.8 yrs) achieved 175 blastocysts that were cultured in closed system using the time-lapse photography (EmbryoScope™ incubator) in our PGT-M program, from August 2017 to March 2020. The recorded films were analysed and revealed that 112 (64%) embryos expel cell debris/ fragments outside the intact embryo (Fig. 2 and Additional file 1).

Eight patients (mean women age 32.4 + 3.2 years, range 27–37 years) undergoing IVF/ICSI cycle for PGT-M, donated 11 high-quality blastocysts, which were found to be affected following Day-3 blastomere biopsy. The blastocyst morphological grading [23], their appearances, and the CGH results of the blastocysts and their corresponding cell debris/fragments are presented in Table 1.

**Table 1:**
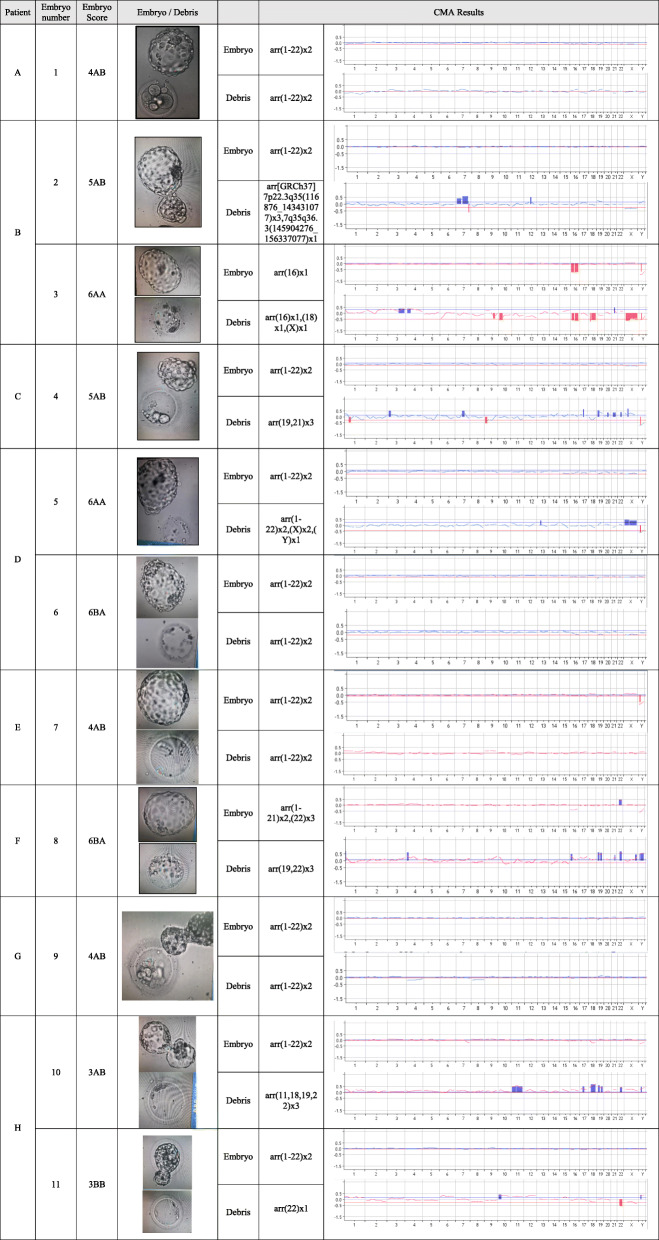
The appearance and CGH results of the blastocysts and their corresponding cell debris/fragments

Seven out of the 11 pairs of blastocysts/cell debris revealed discordant results. Of the 9 euploid blastocysts, the cell debris of four were euploid (embryos #1,6,7,9), four others demonstrated trisomies 19,21 (embryo #4); trisomies 11,18,19,22(embryo #10); trisomy 7(embryo #2); and monosomy 22(embryo #11), and one was XXY(embryo #5). In the remaining two pairs, one blastocyst was monosomic 16 (embryo #3) and the debris monosomic 16, 18 and X, while the other showed trisomy 22 (embryo #8) and its debris revealed trisomy 22, in addition to trisomy 19.

## Discussion

In the present study of extended embryo culture, we are witness of human embryos self-correction mechanism, discovered by their ability to eliminate/expel abnormal blastomeres as cell debris/fragments (Fig. 2 and Additional file 1). Seven (63.6%) out of the 11 pairs of blastocysts expelled cell debris with additional chromosomal rearrangements. Moreover, of the 9 euploid blastocysts, 5 (55.5%) expelled aneuploidy debris.

Women’s fecundity decreases gradually with increasing age, coincident with increased embryonic aneuploidy and spontaneous miscarriages rates [26]. These observations have led to the attractively logical, but still equivocal hypothesis of PGT-A, that the transfer of only euploid embryos should improve IVF outcomes [27, 28]. Even more perturbing, is the reported efficacy of noninvasive PGT-A (niPGT-A) in the spent culture media of human blastocysts by analyzing the cell-free DNA. Huang et al. [29] have reported a zero false-negative rate for niPGT-A, with both the positive predictive value and specificity found to be much higher than TE biopsy PGT-A. Since the concordance rates for both embryo ploidy and chromosome copy numbers were higher for niPGT-A, they suggested that niPGT-A is less prone to errors associated with embryo mosaicism and is more reliable than TE-biopsy PGT-A.

The observed ability of human embryos to self-correction doubts many invasive and ni-PGT-A at the blastocyst stage, rendering high rate of false positive (discarding “good” embryos), by identifying the cell-free DNA originated from the expelled cell debris as aneuploidy/mosaic blastocyst. In their editorial, Gleicher and Barad [30] have provided further explanation to the pitfalls of niPGT-A, e.g. “TE and ICM are assumed to leak into spent media, but TE is in direct contact with medium and ICM is not”. Our observation of human embryo ability to self-correction and expulsion of aneuploidy cells to the culture media, and the reported incongruity between TE and ICM biopsies [12], further highlights the unreliability of ni-PGT-A.

Huang et al. [29] have further speculated that “at least for the euploid embryos, the leakage of DNA from the euploid cells outweighs that of the apoptotic aneuploid cells; otherwise, niPGT-A would not be able to report the euploid embryos successfully”. This assumption was not confirmed in our study, but the opposite. Five out of the 9 euploid whole blastocysts examined revealed different CGH results in their expelled cell-debris: trisomies 19,21; trisomies 11,18,19,22; trisomy 7; monosomy 22, and XXY.

Low-level mosaicism is a common feature of early human development. A recently published study on single-cell genomic data revealed widespread mosaic aneuploidies, with 80% embryos harboring at least one putative aneuploid cell, with no significant enrichment of aneuploid cells in the TE compared to the ICM [31]. These observations conclusively demonstrate that mosaicism in blastocyst-stage embryos is basically a normal physiological phenomenon that can be found in almost all embryos [14, 31].

**In conclusion,** the present study sheds more light on human embryogenesis and its capability for self-correction. Before adopting any future diagnostic procedure aiming to improve embryo selection during an IVF cycle, the aforementioned discussed physiological/embryological observations should be considered in an attempt to improve the test validity.

## Supplementary information

**Additional file 1.** Time-lapse EmbryoScope™ photography of embryo expelling cell debris/cell fragments within the zona pellucida (https://youtu.be/3RNUJ4iW0IE).

## Data Availability

The datasets used and/or analysed during the current study are available from the corresponding author on reasonable request.
