# Supplementary material for: Do human embryos have the ability of self-correction?
Source: Reprod Biol Endocrinol. 2020 Oct 6;18:98. doi: 10.1186/s12958-020-00650-8 (PMC7539487; doi:10.1186/s12958-020-00650-8)
Supplement: Supplementary file 1 — Additional file 1. Time-lapse EmbryoScope™ photography of embryo expelling cell debris/cell fragments within the zona pellucida (https://youtu.be/3RNUJ4iW0IE). [file 12958_2020_650_MOESM1_ESM.docx]

**Figure 2b:** Time-lapse EmbryoScope™ photography of embryo expelling cell debris/cell fragments within the zona pellucida (<https://youtu.be/3RNUJ4iW0IE>).
